# The effect of human gene therapy for *RPE65*-associated Leber’s congenital amaurosis on visual function: a systematic review and meta-analysis

**DOI:** 10.1186/s13023-020-1304-1

**Published:** 2020-02-14

**Authors:** Xue Wang, Chaofeng Yu, Radouil T. Tzekov, Yihua Zhu, Wensheng Li

**Affiliations:** 1 Aier School of Ophthalmology, Central South University, Changsha, China; 2 Shanghai Aier Eye Hospital, 1286 Hongqiao Road, Shanghai, China; 3 Department of Ophthalmology, University of South Florida, Tampa, Florida, USA; 4 First Affiliated Hospital of Fujian Medical University, Fuzhou, China

**Keywords:** Gene therapy, Leber’s congenital Amaurosis, Visual function, Meta-analysis

## Abstract

**Background:**

*RPE65*-associated LCA (*RPE65*-LCA) is an inherited retinal degeneration caused by the mutations of *RPE65* gene and gene therapy has been developed to be a promising treatment. This study aims to evaluate the association between changes in visual function and application of gene therapy in patients with *RPE65*-LCA.

**Methods:**

Several databases (PubMed, Cochrane Library, and Web of Science) were searched for results of studies describing efficacy of gene therapy in patients with *RPE65*-LCA. Six studies, which included one randomized and five prospective non-randomized clinical trials, 164 eyes met our search criteria and were assessed.

**Results:**

The BCVA significantly improved in treated eyes at 1 yr post treatment by − 0.10 logMAR (95% CI, − 0.17 - -0.04; *p* = 0·002), while there was no significant difference at 2–3 years post treatment (WMD: 0.01; 95% CI, − 0.00 - 0.02; p = 0·15). FST sensitivity to blue flashes also improved by 1.60 log (95% CI, 0.66–2.55; *p* = 0.0009), but no significant difference to red flashes (WMD: 0.86; 95% CI, − 0·29–2.01; *p* = 0.14) at 1 yr. There was no significant difference in central retinal thickness at 1 yr, but central retina in treated eyes appeared thinner at 2–3 years post treatment by 19.21 μm (95% CI, − 34.22 - -4.20; *p* = 0.01).

**Conclusions:**

Human gene therapy is a pioneering treatment option for *RPE65*-LCA. Although its efficacy appears to be limited to less than 2 yrs after treatment, it carries the potential for further improvement and prolongation of efficacy.

## Background

Leber’s Congenital Amaurosis (LCA) is a heterogeneous group of eye diseases with mostly autosomal recessive inheritance, characterized with nystagmus and severely decreased visual acuity in early infancy and complete blindness by the third-to-forth decade of life [1]. *RPE65*-associated LCA (*RPE65*-LCA) is associated with mutations of the *RPE65* gene encoding the retinoid isomerohydrolase in the retinal pigment epithelium (RPE), which result in rod-cone type retinal dystrophy [2] [3]. As a cutting-edge approach, human gene therapy was developed to compensate genetic deficiency and improve visual function of *RPE65*-LCA as early as 2008 [4–6]. Since then several studies reported that *RPE65* gene therapy could improve visual function in *RPE65*-LCA; however, the overall level of efficacy remains somewhat uncertain and variable. Therefore, we systemically searched and analyzed the published literature in order to gain a better understanding of the effectiveness of human gene therapy on visual function in *RPE65*-LCA.

## Methods

This meta-analysis was confirmed to the recommendations of the Cochrane Handbook and reported according to the PRISMA reporting guidelines for meta-analysis and systematic reviews [7]. The PRISMA checklist was provided in Additional file 1: Table S1.

### Search methods

Online electronic databases (PubMed, Web of Science, and the Cochrane Library) were searched in November 2018 without restriction to region, date, language or publication types. The following MeSH terms and their combinations were used in [Title/Abstract]: Leber Congenital Amaurosis, RPE65 and gene therapy. Additionally, the web-based resource Clinical trials.gov (https://clinicaltrials.gov) was used to complement the searches of the reference lists of all retrieved studies. When multiple published articles described the same population, the most recent or complete report was used.

### Inclusion and exclusion criteria

All randomized controlled trials (RCTs) and observational studies that reported results of human gene therapy for RPE65-LCA, and that had at least one quantitative outcome of visual function mentioned, were included; however, review articles, meeting abstracts and pre-clinical studies were excluded.

### Study selection

**Fig. 1:**
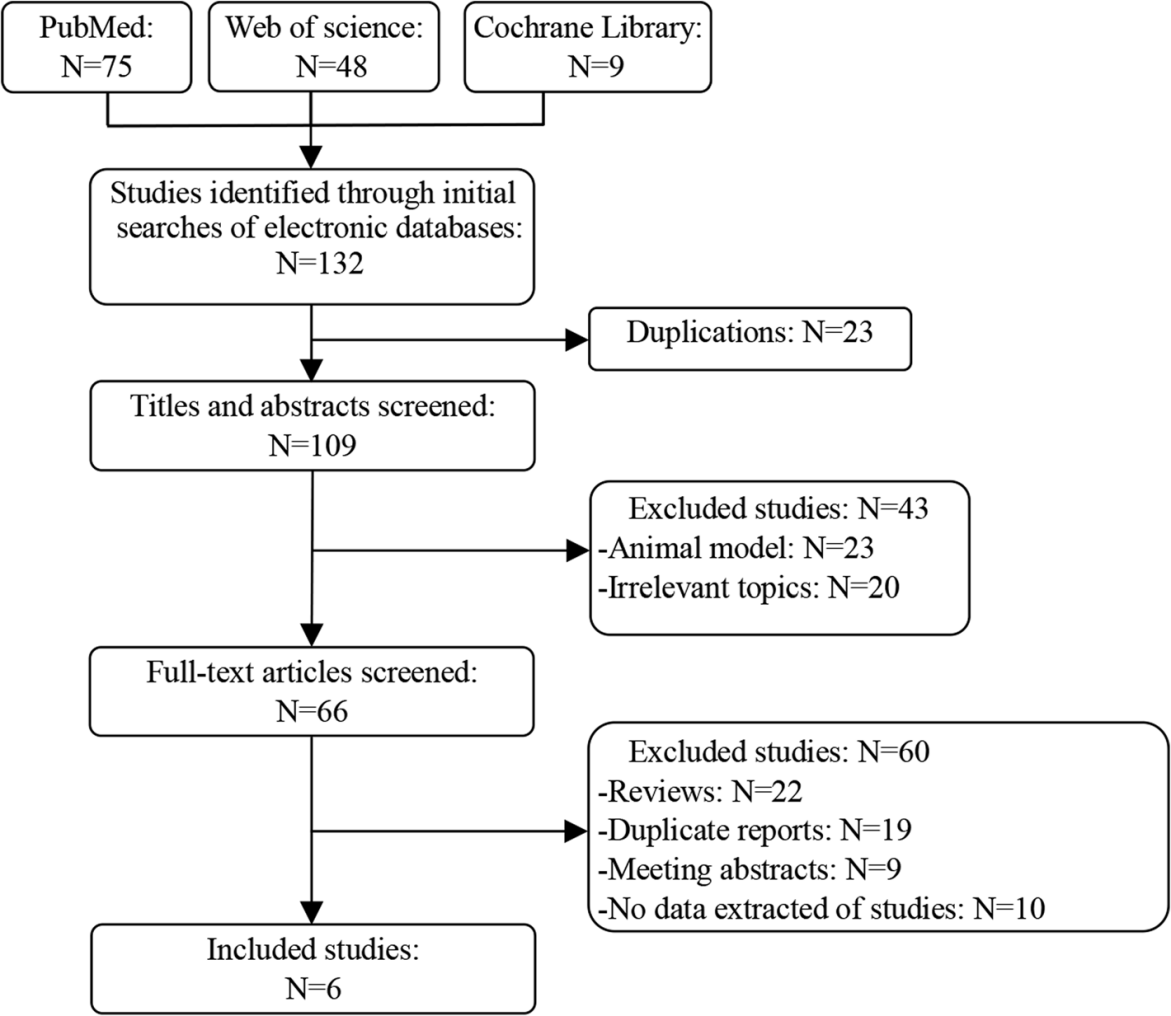
Flow diagram of studies identified, included and excluded. 132 publications were identified from the literature research. Of these, 23 duplicates were removed, 23 were animal models, 20 were irrelevant topics, 22 were reviews, 19 were duplicate reports, 9 were meeting abstracts, and 10 were studies with not relevant data reported

Figure 1 shows a flow chart of the selection process used to identify relevant studies. Data of included studies were extracted and summarized independently by two authors (X.W. and C.Y.). Any disagreement was resolved by the third expert (W.L.). The main outcomes were best-corrected visual acuity (BCVA), and the other outcomes were central retinal thickness, and Full-field Light Sensitivity Threshold (FST) Testing.

### Data collection and risk of bias assessment

Studies were rated for the level of evidence provided according to criteria by the Centre for Evidence-Based Medicine (Oxford, UK). The methodological quality of all cohort studies was assessed by the Newcastle-Ottawa scale (NOS) [8], which consists of three factors: patient selection, comparability of the study groups, and assessment of outcome (Additional file 1: Table S2). A 10-point scale was used and a score of 0–9 was allocated to each study except the RCT. The one randomized trial was considered to be of higher quality for the purposes of this analysis. Two reviewers (X.W. and C.Y.) assessed the quality of the studies. Any discrepancies were resolved by a third reviewer (W.L.). Randomized clinical trials (RCTs) and cohort studies achieving a score of seven or more points were considered to be of high quality.

### Data synthesis and analysis

All analyses were performed using Review Manager 5.3 (Cochrane Collaboration, Oxford, UK). The weighted mean difference (WMD) and risk ratio (RR) were used to analyze continuous and dichotomous variables, respectively. All results were reported with 95% confidence intervals (CIs). If continuous data were presented as means and range values, the standard deviation (SD) were calculated using the technique described by Hozo et al. [9]. Heterogeneity between studies was assessed by the χ^2^ and I^2^ statistic. The random-effects model was used if the *p* value was less than 0·1, otherwise, the fixed-effects model was reported [10].

Subgroup analyses were performed to compare BCVA at baseline of less than or more than 1.3 logMAR based on a recent study which reported that BCVA improvement was different between eyes with a baseline acuity at more than 1.3 logMAR (~ 20/400) vs. eyes with lower baseline BCVA [11]. Funnel plots were used to screen for potential publication bias.

## Results

### Included studies

Overall, 132 publications were identified using the predefined search algorithm (Fig. 1). Of these, 66 publications were related to our research topic. The breakdown of these publications was as follows: 22 were reviews, 19 were duplicate reports, 9 were meeting abstracts, and 10 were studies with not relevant data reported. Six studies with 164 eyes (82 patients) were included in the final analysis [12–17]. Agreement between the two reviewers was 100% for study selection and 83% for quality assessment of trials after examination of references listed for studies. Study outcomes were shown in Table 1.

### Characteristics of included studies

The characteristics of included studies are shown in Table 2. Among the included studies, only one was a RCT [16], while five were prospective clinical trials [12–15, 17]. These studies included eyes with wide range of baseline BCVA, ranging from light perception only to 0.31 logMAR (~ 20/41). Of these, three studies had enough data to allow stratification by baseline BCVA and a subgroup analysis was carried based on the baseline BCVA [13, 15, 17]. The remaining three studies had insufficient data for this type of sub-analysis. In terms of follow-up period of observation, the effectiveness of more than one result related to visual function assessment were recorded at only the 1-year post-treatment interval for one study [16]; while two studies had recorded the effectiveness only at 2–3 years [12, 15]. The remaining three studies had results available at both 1 year and 2–3 years post-treatment [13, 14, 17].

**Table 1:** Results of meta-analysis comparison of treated and untreated group

Outcomes of interest	Studies, no.	Treated Eyes, no.	Untreated Eyes, no.	WMD/RR (95%CL)	P value	Study heterogeneity			
						χ^2^	df	I^2^, %	P value
Main outcomes									
Mean BCVA change at 1 year	4	57	41	−0.10 (−0.17 to −0.04)	0.002	8.88	5	44	0.11
Mean BCVA change at 2–3 years	4	31	32	0.01 (−0.00 to 0.02)	0.15	6.80	5	27	0.24
Other outcomes									
FST sensitivity to red flashes	2	37	21	0.86 (−0.29 to 2.01)	0.14	18.32	1	95	<0.0001
FST sensitivity to blue flashes	2	37	21	1.60 (0.66 to 2.55)	0.0009	3.25	1	69	0.07
Change in central retinal thickness at 1 year	2	52	30	−11.68 (−32.49 to 9.14)	0.27	4.21	1	76	0.04
Change in central retinal thickness at 2–3 years	2	18	18	−19.21 (−34.22 to −4.20)	0.01	0.38	1	0	0.54

**Table 2:** Characteristics of included studies

Studies	Age^&^ (years)	Type of study	Number of eyes enrolled		Eye stratified by BCVA		Follow-up (months)	Outcome measures used	Quality score
			Treated	Untreated	Better BCVA*	Worse BCVA*			
Jacobscon.et al. 2012 [12]	20	P	15	15	11/11	4/4	36	CRT, FST	7.5
Testa.et al. 2013 [13]	20	P	5	5	2/3	3/2	12–36	BCVA	6.5
Bainbridge.et al. 2015 [14]	14	P	12	12	11/11	1/1	12–36	BCVA, CRT	7.5
Weleber.et al. 2016 [15]	25	P	12	12	7/7	5/5	24	BCVA	7.5
Russell.et al. 2017 [16]	15	RCT	40^#^	18^#^	32^#^/16^#^	8^#^/2^#^	12	BCVA, CRT, FST	RCT
Le Meur.et al. 2018 [17]	24	P	9	9	5/7	4/2	12–36	BCVA	7.5

& - average age at recruitment (years)

# - bilateral administration

*Better BCVA - baseline BCVA better than 1.3 logMAR; *Worse BCVA - baseline BCVA worse than 1.3 logMAR

Legend: P – prospective clinical trial; RCT – randomized controlled clinical trial; BCVA - best-corrected visual acuity; CRT - central retinal thickness; FST - full-field sensitivity threshold

### Methodological quality of included studies

The quality of included studies was relatively high, with an average score of 7.3. True randomization was used in only one RCT [16]. For the RCT where treatment was administered bilaterally, visual function parameters were averaged for both eyes and recorded as one value used in the statistical analysis, while in the five prospective studies treatment was administered unilaterally and visual function was assessed and reported bilaterally. None of the prospective studies provided information about allocation sequence generation and concealment or about the blinding method. Matching criteria between two groups were variable. Apart from the RCT, the eye of each participant with the poorer visual acuity was selected as the study eye, and the contralateral eye served as an untreated control, therefore, the BCVA at baseline was not matched.

### Main outcomes

#### Mean change in BCVA

**Fig. 2:**
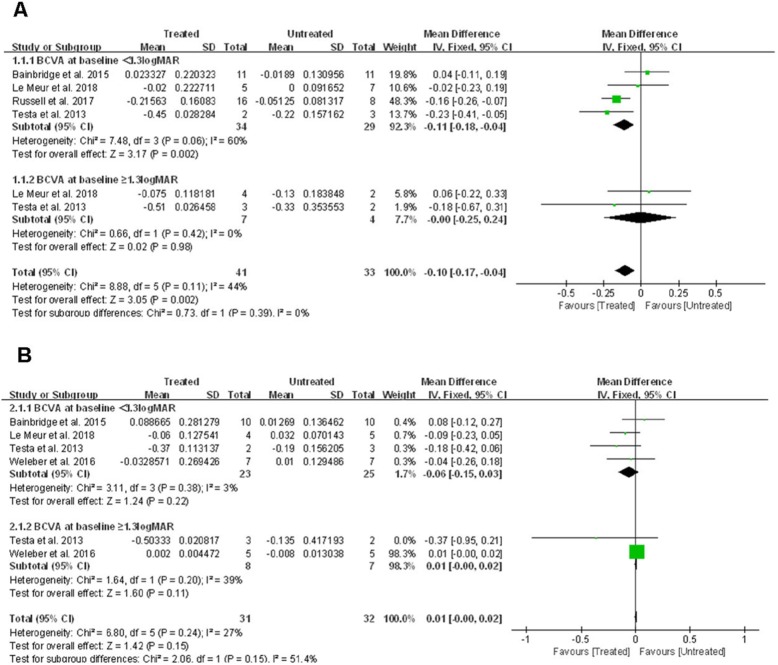
Forest plot and meta-analysis of mean BCVA improvement on the Early Treatment Diabetic Retinopathy Study eye chart. (A) Mean BCVA improvement in treated eyes compared to untreated eyes at 1 year. (B) Mean BCVA improvement in treated eyes compared to untreated eyes at 2–3 years. BCVA: best-corrected visual acuity, LogMAR: Logarithm of minimum angle of resolution

BCVA was measured using the Early Treatment Diabetic Retinopathy Study (ETDRS) method, and the acuity was scored as the number of letters read after adjusting for distance and expressed as logMAR. Pooling the data from four studies [13, 14, 16, 17] that assessed BCVA in 98 eyes from 49 patients showed that BCVA improved significantly in treated eyes compared to untreated eyes at 1 yr post treatment by − 0.1 logMAR (95% CI, − 0.17 to − 0.04; *p* = 0.002), with no significant heterogeneity between studies (χ^2^ = 8.88, df = 5, *p* = 0.11; I^2^ = 44%) (Fig. 2a).

At later follow-up time points (2–3 years post treatment), four studies [13–15, 17] including 63 eyes from 32 patients had data allowing estimation of BCVA change. The pooled data showed no significant difference between treated vs. untreated eyes (WMD: 0.01; 95% CI, − 0.00 to 0.02; *p* = 0.15), with no significant heterogeneity between studies (χ^2^ = 6.80, df = 5, *p* = 0.24; I^2^ = 27%) (Fig. 2b).

#### Subgroup analysis

When treated eyes were compared with untreated eyes in patients with baseline acuity better than 1.3 logMAR, it appeared that BCVA in treated eyes improved significantly by − 0.11 logMAR (or more than one line on the ETRDS chart) at one-year post-treatment (95% CI, − 0.18 – − 0.04; *p* = 0.002). However, there was no significant difference in BCVA change at 2–3 years of follow-up (WMD: -0.06; 95% CI, − 0.15 – 0.03; *p* = 0.22).

Furthermore, when BCVA in eyes with a baseline acuity of more than 1.3 logMAR were compared, there was no significant difference between mean BCVA change at the 1 yr post treatment visit (WMD: -0.00; 95% CI, − 0.25 – 0.24; *p* = 0.98), and also at 2–3 years post treatment (WMD: 0.01; 95% CI, − 0.00 – 0.02; *p* = 0.11).

### Other outcomes

#### Change in FST sensitivity

**Fig. 3:**
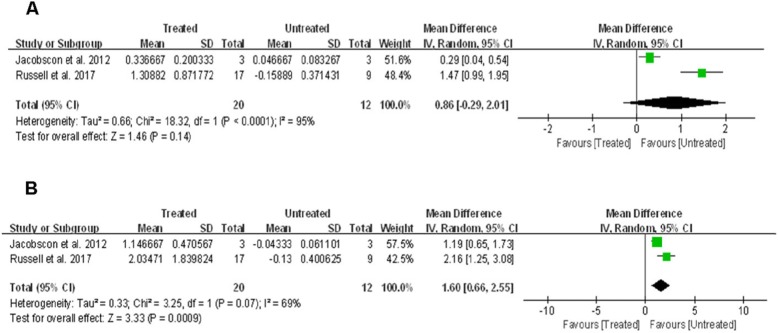
Forest plot and meta-analysis of FST sensitivity improvement. (A) FST sensitivity improvement to red flashes in the treated eyes vs. untreated eyes. (B) FST sensitivity improvement to blue flashes in the treated eyes vs. untreated eyes. FST: Full-field Light Sensitivity Threshold

FST was performed using a LED-based Ganzfeld stimulator, red and blue stimuli were used to probe differential effects on cone versus rod photoreceptors [18]. FST sensitivity to red flashes data were available for 58 eyes (29 patients) across two studies [12, 16]. The mean change was higher by ~ 0.86 log in the treated eyes vs. untreated eyes, but the difference was not statistically significant (95% CI, − 0·29–2.01; *p* = 0.14), with significant between-study heterogeneity (χ^2^ = 18.32, df = 1, *p*<0.0001; I^2^ = 95%) (Fig. 3a).

Additionally, FST sensitivity to blue flashes data were also available for 58 eyes (29 patients) across two studies [12, 16]. The mean change was significantly higher in the treated eyes vs. untreated eyes by ~ 1.60 log (95% CI, 0.66–2.55; *p* = 0.0009), with no significant between-study heterogeneity (χ^2^ = 3.25, df = 1, p = 0·07; I^2^ = 69%) (Fig. 3b).

#### Change in central retinal thickness

**Fig. 4:**
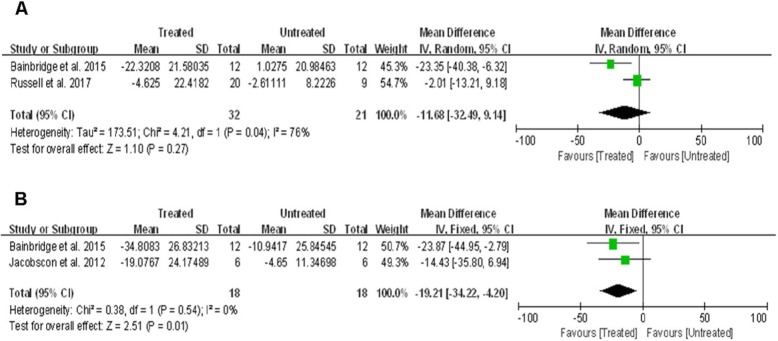
Forest plot and meta-analysis of retinal degeneration at 1 year (**a**) and 2–3 years (**b**) post-treatment

Total thickness of central retinal was measured using spectral domain optical coherence tomography. Two studies [14, 16] that assessed 82 eyes from 41 patients reported on change in central retinal thickness at the 1 yr visit. Despite some tendency for more pronounced thinning in the central retina of treated eyes, the analysis showed no significant difference between the treated vs. untreated eyes (WMD: -11.68; 95% CI, − 32.49 – 9.14; *p* = 0·27), with significant between-study heterogeneity (χ^2^ = 4.21, df = 1, p = 0·04; I^2^ = 76%) (Fig. 4a).

At later follow-ups (2–3 years after treatment), two studies [12, 14] including 36 eyes from 18 patients assessed central retinal thickness, which showed that the change in central retinal thickness was on average ~ 19.21 μm lower in treated eyes compared to untreated eyes (95% CI, − 34.22 – − 4.20; p = 0·01), with no significant between-study heterogeneity (χ^2^ = 0.38, df = 1, *p* = 0·54; I^2^ = 0%) (Fig. 4b).

### Publication bias

**Fig. 5:**
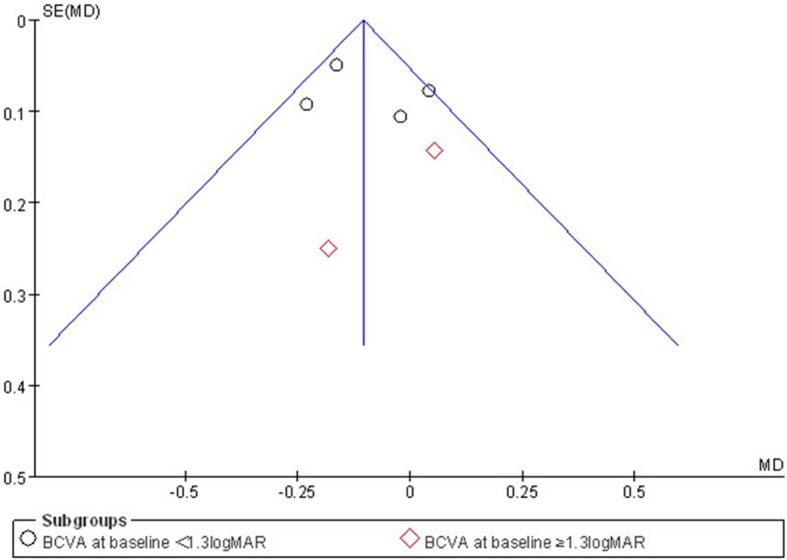
Funnel plots illustrating meta-analysis of mean BCVA improvement at 1 year. SE = standard error; MD = mean deviation

Figure 5 shows a funnel plot of the studies included in this meta-analysis that reported mean change in BCVA at year one visit. All studies lie inside the 95% CIs, with an even distribution around the vertical, indicating no obvious publication bias.

## Discussion

This meta-analysis summarizes the results from one randomized and five prospective clinical trials, including 164 eyes from 82 patients and comparing visual function of treated vs. untreated eyes. It showed that, in terms of improvement of best-corrected visual acuity and full-field light sensitivity threshold to blue flashes, gene therapy was effective up to 2 yrs post treatment. However, the improvement in BCVA was not sustainable and the data were not available in FST sensitivity beyond 2 yrs post treatment.

Recent studies indicate a good safety profile for this type of treatment, with no difference at the rate of serious ocular adverse events even at 5 yrs post treatment [11]. However, there are some indications that the retinal degeneration associated with the disease may be occurring faster in treated eyes compared to non-treated eyes. Thus, some tendency for thinning was apparent even at 1 yr post treatment, although the difference was not statistically significant. More importantly, at later follow-up time points (2–3 years post treatment), the analysis showed significant thinning of the central retina in treated eyes vs. non-treated eyes. The average difference in thinning of ~ 19 μm at 2–3 years between the two groups appears to be also clinically significant as it translates to ~ 8% difference in thickness, assuming ~ 250 μm average central retinal thickness. Although the reason for this deference in central retinal thickness at more than 2 years post treatment is currently unknown, it can be hypothesized that temporary retinal edema or detachment caused by the subretinal injection had some limited, but measurable and lasting damage to the retinal structure which ended up thinner upon resolving the edema or detachment. Besides, the eyes selected for treatment with gene therapy had relatively worse baseline visual acuity vs. untreated eyes, with an average difference of 0.33 logMAR (or more than 3 lines on the ETDRS chart), which might indicate a more advanced stage of the disease.

The effects of any human gene therapy are complex and multifaceted. The results from this meta-analysis indicate that gene therapy does not slow visual function loss in the long term (beyond 2 yrs). Visual function loss in LCA is caused by a combination of biochemical chromophore deficiency and progressive degeneration of photoreceptor cells [19], but gene therapy appears to address only the biochemical chromophore deficiency under the premise of a certain proportion of surviving photoreceptor cells. However, continuing loss of visual function from the ongoing retinal degeneration can still occur following initial improvement from gene therapy. Thus, in order to improve future efficacy, it may be beneficial to administer combinatorial agents supplementing the gene therapy with the goal to prevent further loss of retinal cells. A study by Cideciyan et al. demonstrates advancing retinal degeneration despite visual improvement after gene therapy for RPE65-LCA, and suggests the need for a combinatorial strategy to improve vision function in short term but also to slow retinal degeneration in the long term [20]. Potential agents to be considered as part of the combinatorial therapy could be neuroprotective, prosurvival, antiapoptotic factors or antioxidants. These agents could be a significant factor delaying or preventing continuing cone photoreceptor cell loss observed in animal models of inherited retinal degenerative diseases [21–23], administered either simultaneously or sequentially with gene therapy. One example of such agent could be l-cysteine, as it was recently shown to be neuroprotective for RPE [24, 25]. One downside of the implementation of this approach is that regulatory agencies (e.g. FDA in the US, EMA in Europe, etc.) may require separate studies to evaluate safety and efficacy on each one of these components, unless there is sufficient animal data to support additive effects.

In the advanced or endstage of the disease, it is likely that the structure of the outer retina has already undergone considerable damage, and gene therapy might not be able to regenerate most photoreceptor cells, likely damaged beyond repair. Therefore, emerging techniques, such as stem cell transplantation and retinal prosthesis, may be proposed as therapeutic strategies to restore visual function. When analyzing the BCVA data it became apparent that, surprisingly, some contralateral (untreated) eyes showed improvement in BCVA after treatment [12–15]. This phenomenon makes it complicated to estimate the “true” improvement of BCVA as the treatment effect was based on a comparison between treated vs. contralateral (untreated) eyes. Although a small learning effect may contribute to this phenomenon, it is likely that the main cause of this effect is related other factors. One such factor could be a reorganization of the receptive fields of retinal ganglion cells which could be due to efferent influences or even direct retino-retinal connections [26, 27]. Further studies including visual electrophysiological tests would be helpful to understand the origin of this improvement.

In the studies subject to this meta-analysis, visual function was tested in several different ways. One of the methods used was testing FST in dark-adapted eyes by stimulating the retina with red or blue flashes. It is generally accepted that red FST flashes stimulate more effectively the cone population (mainly L-cones), while blue flashes stimulate preferentially the rods (and some S-cones). The FST sensitivity analysis showed that gene therapy improved the rod function by ~ 137% and cone function by ~ 89% vs. baseline in the treated eyes at 1 yr post treatment, but there was no significant difference between treated vs. untreated eyes at that time point in sensitivity to red flashes (reflecting dark-adapted cone function). This finding suggests that human *RPE65* gene therapy has a stronger positive effect on rod photoreceptor function. Besides, there may be an additional factor influencing the observed discrepancy between rod and cone function improvement. Both types of stimulation stimulated the total retinal area, however, the subretinal injection was generally limited to the macula, which has a relatively small size compared to the total retinal area (< 3%). Although the cone density is lower in the periphery, the overall number of cones in the periphery is much higher compared to the number of cones in the macula (> 10 times) [28]. Therefore, even if the function of the central cones has improved (as suggested by the improvement in BCVA), this positive change may be too small to be reflected in the global response from all cones. A better parameter to detect visual function improvement after gene therapy would be visual field testing (kinetic perimetry, static automated perimetry, microperimetry, etc.). However, the visual field outcome measures used in the studies analyzed here were too varied and prevented us from conducting a rigorous meta-analysis. Nevertheless, from Russel et al. and Weleber et al., it was implied that the fovea could be particularly vulnerable to degeneration in LCA [15, 16]. Russel et al. concluded that Humphrey macula sensitivity threshold was increased in the intervention group, but Humphrey foveal sensitivity threshold was not. And Weleber et al. concluded that V_30_ increased in 6 patients, while V_TOT_ increased in 5 patients vs. kinetic visual field area improved in only 3 patients in the treated eye. It is possible that foveal and extrafoveal cones do not have the same relationship with RPE apical processes and contributions of the chromophore required from retinal and RPE visual cycle pathways may also differ between foveal and extrafoveal cones [29, 30].

Between-study heterogeneity was not significant for most of the outcomes except for FST sensitivity to red flashes and the change in central retinal thickness at 1 yr. The difference in sample size, follow-up time points, and bilateral or unilateral administration among the studies might have contributed to the significant between-study heterogeneity for these parameters. The random-effects model was used to reduce the effect of heterogeneity but did not eliminate it completely.

This meta-analysis has some limitations that need to be taken into account. The main limitation is the insufficient number of RCTs (only one available) resulting in inadequate random sequence generation and blinding leading to an increased risk of bias. However, it has to be noted that *RPE65*-LCA is a rare disease with an estimated prevalence of about 1:80,000, and the incidence of *RPE65*-LCA would be about 6% of all LCA [31, 32]. Finding patients for such a rare disease is a challenge and is difficult to anticipate an adequate number of RCT trials to appear in the near future. Besides, there was a lack of available data for subgroup analysis based on stratification of the endpoints in the current analysis (except BCVA). However, according to the subgroup analysis of BCVA, the effectiveness of gene therapy with different baseline acuity might be inconsistent. Finally, as enough data at comparable follow-up time points was lacking, the analysis could not accurately assess time-dependent effectiveness beyond the relatively crude separation of 1 yr vs. 2–3 yrs of follow-up.

Although all the limitations listed in the previous paragraph, the results appeared relatively homogenous (except FST to red flash and central retinal thickness change at 1 yr visit) and seemed to suggest that results of the meta-analysis were still valid. The recent availability of data related to the visual outcomes of clinical trials using gene therapy for of RPE65-LCA proved fortunate as enough data are now available for an initial evaluation by meta-analytical methods. However, this should be considered only an initial evaluation of this type of therapy, which would become without doubt an important and expanding field of future clinical research.

## Conclusions

In summary, this meta-analysis indicates that *RPE65* gene therapy is associated with an improvement of BCVA and FST sensitivity to blue flashes in the short-term (up to 2 yrs post treatment). Visual function appears to be equivalent in the longer term (2 yrs and beyond) in terms of change in the visual function measures analyzed here like BCVA. The inherent limitations of included studies may have an impact on precise conclusion several important aspects of efficacy. Only larger-scale, well-designed RCTs would be able to clarify these aspects and provide further insight and guidance into the benefits and risks associated with *RPE65*-LCA gene therapy.

## Supplementary information

**Additional file 1: Table S1.** PRISMA checklist. **Table S2.** Risk of bias in cohort studies using Newcastle Ottawa scale (NOS).

## Data Availability

Data are available from the authors upon request.

## Abbreviations

BCVA: Best-corrected visual acuity
CI: Confidence interval
CRT: Central retinal thickness
ETDRS: Early Treatment Diabetic Retinopathy Study
FST: Full-field Light Sensitivity Threshold
LCA: Leber’s Congenital Amaurosis
MD: Mean deviation
NOS: Newcastle-Ottawa scale
RCT: Randomized controlled trial
RPE: Retinal pigment epithelium
RR: Risk ratio
SD: Standard deviation
SE: Standard error
WMD: Weighted mean difference
